# Danger of Arsenical Injections in Subjects Intended for Dissection

**Published:** 1846-01

**Authors:** 


*Danger of Arsenical Injections in Subjects intended for Dissection.* By M. Benoit.—As arsenical injections are very often used now to preserve bodies for dissection, it is right to be aware of the dangers resulting from the practice. At a *concours* held lately at Montpelier, six bodies were given for dissection, five of which were injected with an arsenical solution of the strength of about 900 grains of arsenious acid to about 2¼ pounds of water. The five gentlemen to whom these injected subjects were allotted were attacked with severe symptoms of arsenous poisoning, as drowsiness, colic, diarrhœa, vomiting. All, too, were affected with a severe and continuous lancinating pain at the extremities of the fingers. The pain had its chief seat in the bulb of the finger and the circumference of the nail. The painful part was visibly swollen, and an appearance resembling ecchymosis was perceived through the nails, which were afterwards cast off. These five individuals were also troubled with uneasy tremors of the upper extremities. They all recovered. It is asked whether these symptoms, especially the pains in the fingers, were produced by the direct absorption of the poison, or by breathing the arseniuretted hydrogen, which it has been ascertained animal bodies exhale when preserved by means of arsenic? Perhaps both causes had their share in the production of the symptoms.—*Ed. Med. and Surg. Jour.* from *Jour. de Pharm.*

